# Impacts of Climate Change and Related Weather Events on the Health and Wellbeing of Culturally and Linguistically Diverse Communities: A Systematic Review

**DOI:** 10.1007/s40615-025-02527-1

**Published:** 2025-06-26

**Authors:** Sitasma Sharma, Olga Anikeeva

**Affiliations:** School of Public Health, Faculty of Health and Medical Sciences, The University of Adelaide, Adelaide, South Australia Australia

**Keywords:** Climate change, Culturally and linguistically diverse (CALD) communities, Health impacts, Extreme weather events, Systematic review

## Abstract

**Background:**

Vulnerable populations such as culturally and linguistically diverse communities (CALD), ethnic minorities and racial groups face a disproportionate burden of climate change–related health impacts due to a combination of socio-cultural and economic factors, geographic vulnerabilities and health disparities. This review synthesised the existing evidence on the health and wellbeing impacts of climate change and related weather events among CALD communities.

**Methods:**

A narrative synthesis approach was utilised to conduct a systematic review. Three electronic databases (PubMed, Scopus and Web of Science) were searched, identifying 25 studies for appraisal and synthesis. Studies published in the English language from January 2010 to March 2024 were included in the review.

**Results:**

The reviewed studies, mostly carried out in the USA, employed varied study designs, and focused on diverse CALD groups such as migrants, farmworkers and racial and ethnic minorities. The included studies addressed broader and specific climate change–related events, ranging from heat-related impacts and hurricanes to occupational heat exposure. CALD communities were found to be more vulnerable to climate change–related negative physical and mental health issues, further exacerbated by poor living conditions, limited access to healthcare, and cultural and language barriers.

**Conclusion:**

Future efforts by governments, healthcare agencies, employers and research institutions should prioritise multilingual risk communication strategies, providing culturally appropriate health education and healthcare access, housing improvements and the investigation of long-term health impacts of climate change and coping mechanisms adopted among CALD populations.

**Supplementary Information:**

The online version contains supplementary material available at https://doi.org/10.1007/s40615-025-02527-1.

## Introduction

The increasing frequency, severity and duration of weather extremes driven by climate change pose a global challenge with overarching consequences for human health and wellbeing [1, 2]. It is widely recognised that vulnerable and marginalised groups are disproportionately affected by the adverse impacts of climate change [3, 4]. Among these groups, culturally and linguistically diverse (CALD) populations often face unique challenges that increase their susceptibility to climate-related health issues [4–6]. Various definitions of CALD have been adopted around the world. In this study, CALD communities refer to individuals or groups such as migrants, migratory seasonal farm and agricultural workers, disaster crisis migrants and climate refugees, ethnic minorities and racial groups, refugees, undocumented border crossers and those who self-identify as having diverse cultural backgrounds, ethnicities and languages [7].

While there is strong scientific evidence demonstrating the adverse impacts of climate change and extreme weather events on global health, limited attention has been paid to how these impacts are experienced by CALD groups, and significant gaps remain in understanding how they are uniquely affected [2, 8–10]. This gap is particularly concerning as global temperatures continue to rise and extreme weather events become more frequent and severe. A comprehensive understanding of the unique health impacts on CALD groups is currently lacking and there is an urgent need to identify and prioritise the needs of these populations [9, 11]. Therefore, synthesising available evidence is essential to ensure that no communities are left behind in climate adaptation efforts.

The Intergovernmental Panel on Climate Change (IPCC) Sixth Assessment Report highlighted that human-induced increases in greenhouse gases have and will continue to lead to more frequent, widespread and severe extreme weather events, such as heatwaves, hurricanes, cyclones, wildfires, floods and droughts [8]. These events are projected to increase health impacts and risks such as premature deaths, heat-related illnesses, mental health issues, respiratory illnesses, cardiovascular diseases, allergies, waterborne diseases, malnutrition and vector-borne diseases, with estimates of an additional 250,000 deaths per year by 2050 due to conditions including heat stress, malnutrition, malaria and diarrheal diseases [12]. Vulnerable populations, including CALD groups, are particularly at risk of mental health conditions such as anxiety, depression and post-traumatic stress disorder due to increasing extreme weather events followed by displacement and economic instability [3, 13–17].

CALD communities often face compounded vulnerabilities due to systemic barriers such as limited healthcare access, language barriers, inadequate culturally appropriate information and educational resources, and inadequate coping resources [4, 9, 18]. For example, periods of extreme heat can exacerbate pre-existing physical and mental health conditions such as psychiatric conditions, renal, cardiovascular and respiratory diseases, particularly for CALD individuals with limited access to healthcare services and coping resources such as effective cooling equipment and infrastructure [19–21]. Socio-economic disparities further contribute to their risk. For example, they may live in socioeconomically disadvantaged areas with poor quality housing and infrastructure that may be more likely to be damaged by extreme weather or provide little protection from events like extreme heat. These intersecting factors can hinder their ability to adequately prepare for, respond to and recover from extreme weather events [9, 22]. Therefore, it is pivotal to address these issues to better inform the development of targeted, effective and inclusive adaptation strategies and policies that safeguard their health and wellbeing, while promoting greater equity and resilience in the face of climate change’s growing threats.

This study aimed to systematically synthesise and analyse current evidence on the health and wellbeing impacts of climate change and related weather events on CALD groups. The goal was to identify the broader health risks, issues and needs of CALD communities in the context of a changing climate.

## Methods

The review protocol was registered on the International Prospective Register of Systematic Reviews (PROSPERO) (Registration number: CRD42024528377). The Population, Exposure, Comparator, and Outcomes (PECO) framework was used to guide the scope and structure of the review, ensuring a systematic approach [23]. The framework also guided the development and refinement of the search strategy, as well as the inclusion and exclusion criteria for identifying relevant literature.

### Search Strategy

This study followed the Preferred Reporting Items for Systematic Reviews and Meta-Analyses (PRISMA) guidelines to systematically identify and synthesise published literature [24]. A comprehensive search was conducted across three databases: Web of Science, PubMed and Scopus in March 2024. Handsearching was also employed to identify additional relevant articles that may not have been captured in database searches. Search terms included the following: culturally linguistically diverse, CALD, ethnic minorities, migrant, immigrant, refugee, diverse cultural group, climate change, climate change events, extreme heat, heatwave, hot weather, wildfire, hurricane, flood, storms, health, wellbeing, heat-related illnesses, heat stress, mortality, cardiovascular diseases and mental health. Relevant database-specific adjustments were made as needed, for example, Medical Subject Headings (MeSH) terms were used in PubMed. The detailed database-specific search strategies are provided in Supplementary File 1.

### Inclusion and Exclusion Criteria

The inclusion and exclusion criteria for this review are specified in Table 1.

**Table 1:** Review inclusion and exclusion criteria

Inclusion criteria	Exclusion criteria
Studies conducted among adult populations (≥ 18 years old)	Studies conducted among children (< 18 years old) or pregnant women
Studies investigating health and wellbeing outcomes among individuals from culturally and linguistically diverse (CALD) communities	Studies not reporting health and wellbeing outcomes among CALD communities
Studies reporting on the impact of climate change or at least one extreme weather event, such as extreme heat, heatwaves, wildfires, droughts, cyclones, hurricanes, floods and storms	Laboratory and/or experimental studies conducted in heat or climatic chambers
Studies published in English	Studies published in languages other than English for which English-language versions were unavailable
Studies published between January 2010 and March 2024	Studies published before January 2010

Studies focusing on children and pregnant women were excluded because their health outcomes and vulnerabilities can differ substantially from those of the general adult population and require separate, specialised research. Studies published prior to 2010 were excluded to prioritise recent research that better reflects current climate change-related challenges.

**Fig. 1:**
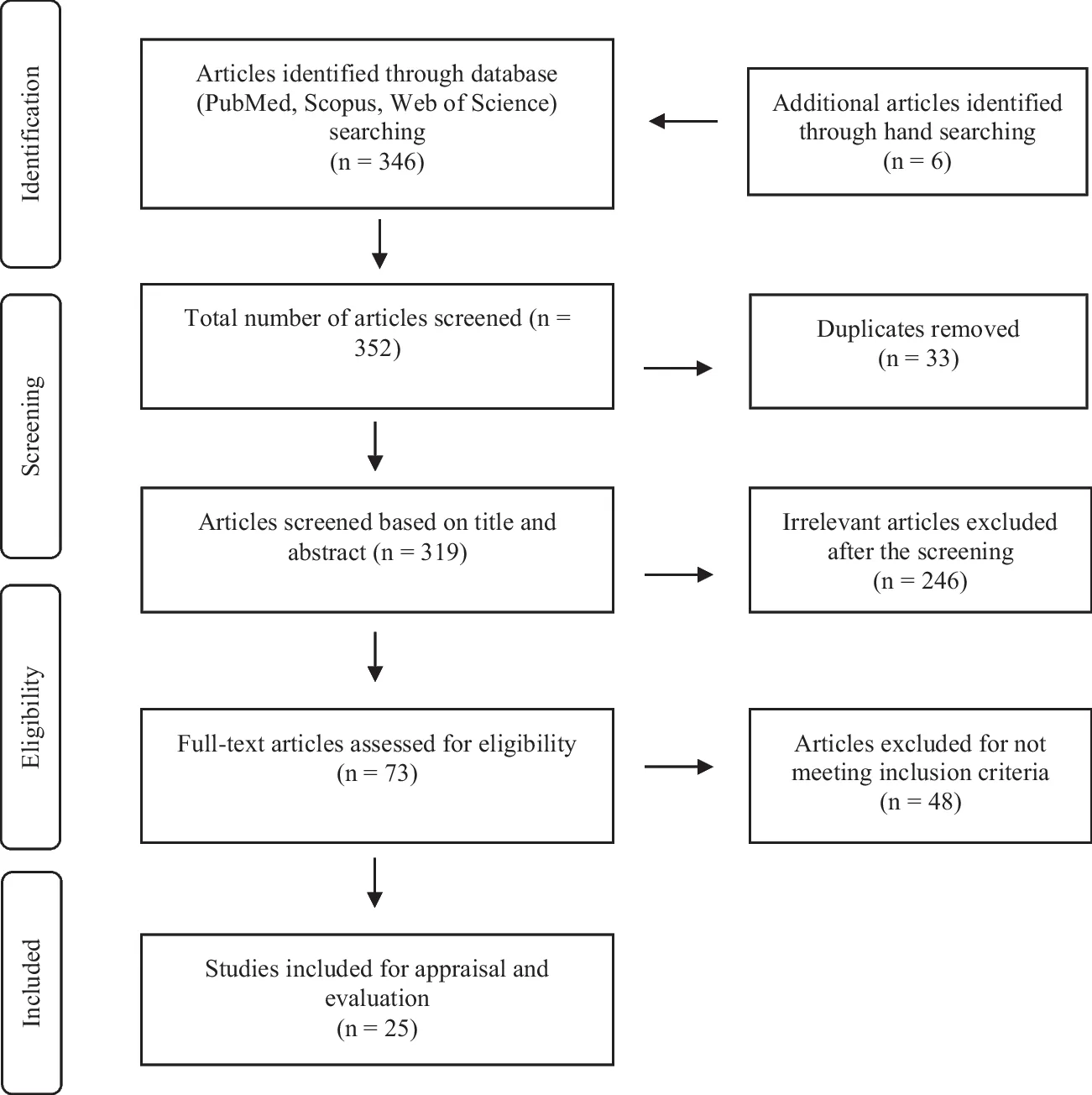
Prisma flowchart diagram

Two reviewers independently screened and assessed articles based on the inclusion and exclusion criteria using COVIDENCE, an online systematic review management tool [25]. Differences in opinion were resolved through discussion. In total, 346 articles were retrieved through database searches and six articles were identified through hand searching. After removing 33 duplicates, 319 articles were screened by title and abstract, leading to the exclusion of 246 irrelevant articles. Among the remaining 73 full-text articles, 48 were excluded due to reasons such as ineligible study types, outcomes, populations, exposures and settings. A total of 25 articles met the inclusion criteria and were included in the review as detailed in Fig. 1.

### Quality Assessment

The quality and rigor of the included studies were assessed using a modified Critical Appraisal Skills Programme (CASP) appraisal tool [26]. Modifications were necessary to accommodate the diverse study designs included in the review. The revised tool allowed for a structured and comprehensive assessment of study quality [26]. The modified CASP tool included ten questions across three domains: validity, results and applicability of the findings for practice and policy.

### Data Extraction, Analysis and Synthesis

Information on study characteristics such as first author name, year of publication, study location, study design, study population, sample size, exposure type, analysis methods and key findings were extracted using a structured spreadsheet. This structured approach ensured all relevant information was captured across included studies.

Narrative synthesis was used to summarise the extracted data, guided by Braun and Clarke’s six steps framework for thematic analysis [27]. Due to the heterogeneity of study designs, this method enabled the synthesis of both qualitative and quantitative findings into a coherent narrative [28]. Both authors were involved in the data extraction, and any disagreements were resolved through discussion. NVivo software was used to facilitate the systematic coding process. During the initial open coding phase, the included articles were reviewed line by line, and key findings were coded into nodes or groups. These nodes were further grouped into broader categories to develop overarching themes. This process allowed for the identification of recurring patterns and key insights across the included studies, resulting in three major themes.

## Results

### Characteristics of Included Studies

The extracted study data are presented in Table 2. The studies employed a range of study designs, including quantitative, qualitative and mixed methods. Of the 25 studies, 40% (*n* = 10) were cross-sectional; four employed mixed methods; four used ecological designs; four were longitudinal/cohort studies; two applied qualitative methods; and one was a case control study. Most of the included studies (*n* = 20) were conducted in various regions of the USA, followed by two in China, and one each in Canada, France and Australia. Six studies specifically focused on migrants affected by hurricanes, while five studies focused on migrant farmworkers. The remaining studies focused on immigrants, refugees, racial and ethnic minorities, and undocumented border crossers. Eight studies examined heat-related impacts; six studies focused on hurricanes such as Harvey, Maria and Katrina; and five addressed occupational heat exposure. One study focused on wildfire impacts, while four considered the broader impacts of climate change.

**Table 2:** Characteristics and key health findings of studies included in the review

Author	Publication date	Country	Study design	Study population	Sample size	Type of exposure	Analyses	Key health findings
Vu and VanLandingham [31]	2011	New Orleans, Louisiana, USA	Pre-post design, longitudinal	Vietnamese immigrants	128	Hurricane Katrina	Descriptive statistics, repeated measures ANOVAs, multivariate analyses	• Health status declined in 7 of 8 SF-36 subscales (physical and mental health) at 1-year post-Katrina • Significant recovery noted by the 2-year mark • Depression symptoms (≥ 5) increased from 34% at baseline to 49% one year after Katrina (*P* = 0.01) • Better social support was linked to improved recovery • Lower acculturation levels were associated with poorer post-Katrina health outcomes
Carl et al. [32]	2019	Puerto Rico, USA	Case control	Puerto Rican migrants	75 controls and 34 migrants	Hurricane Maria	Descriptive statistics, chi-square tests, Fisher’s exact tests, Shapiro–Wilk testing, Kruskal–Wallis tests and Mann–Whitney *U* tests	• Significant differences in severe mental distress ⇒ Spanish-preferring migrants: 30.4% severe mental distress ⇒ English-preferring migrants: 0% severe mental distress
Pham et al. [33]	2023	Houston, Texas, USA	Cross-sectional	Vietnamese American residents	120	Hurricane Harvey	Descriptive statistics, bivariate analyses and multivariable logistic regression modeling	• Mean physical component score: 39.8 (95% CI, 29.7–49.9) • Mean mental component score: 32.6 (95% CI, 27.6–37.6) • Poor physical health: ⇒ More likely in people with ≥ 5 depressive symptoms compared to those with < 5 (OR = 3.04; 95% CI: 1.11–8.29; *P* = 0.03) ⇒ Less likely in those with health insurance than without (OR = 0.25; 95% CI: 0.09–0.71; *P* = 0.01) • Poor mental health: ⇒ More likely in those with serious injury/illness vs no serious injury/illness (OR = 3.34; 95% CI: 1.12–9.94; *P* = 0.03) ⇒ Less likely in people receiving free health care (*P* < 0.05) ⇒ Less likely in people with high vs low levels of acculturation (*P* < 0.05)
Pham et al. [34]	2023	Houston, Texas, USA	Cross-sectional	Vietnamese American residents	120	Hurricane Harvey	Linear regression modeling	• Adverse health outcomes were reported after the disaster • High social support was linked to fewer depressive symptoms • The negative relationship between social support and depressive symptoms persisted, regardless of individuals’ self-rated health after the storm
Montero-Zamora et al. [35]	2023	Puerto Rico, USA	Cross-sectional	Puerto Rican migrants	319	Hurricane Maria	Latent profile analysis (LPA) Multinomial regression modeling	• Low hurricane stress/low cultural stress: 44.7% • Low hurricane stress/moderate cultural stress: 38.7% • High hurricane stress/moderate cultural stress: 6.3% • Moderate hurricane stress/high cultural stress: 10.4% • Worst mental health outcomes: Moderate hurricane stress/high cultural stress • Most important predictor of Poor Mental Health: Postmigration cultural stress
Hodges et al. [36]	2023	Puerto Rico, USA	Cross-sectional	Puerto Rican migrants	319	Hurricane Maria	Descriptive analyses, regression analysis and logistic regression	• PTSD Prevalence: 20.5% Maria migrants • Independent predictors of PTSD: Hurricane trauma and migration-related cultural stressors • Strong links with PTSD: Discrimination and language stress (controlling for hurricane trauma) • Interaction Effects: ⇒ Cultural stress predicts PTSD at low-to-moderate hurricane trauma ⇒ Cultural stress less predictive at high-to-very-high hurricane trauma
Culp and Tonelli [30]	2019	Midwestern Region, USA	Mixed-method-cross-sectional and intensive surveillance (IS) during field trials	Hispanic farmworkers	148 (survey component); 20 (physiological monitoring and field trial component)	Occupational heat exposure	Descriptive statistics such as frequencies, means, standard deviations Analysis of variance (ANOVA) Fisher's exact test Age-adjusted odds ratios	• Heat-related illnesses (HRIs) are largely undocumented • Physiological burden of crop production often overlooked • Uncomfortable Physiological Intensity (PI) score (> 4.0): ⇒ Significantly higher body temperature (mean = 100.05 °F) ⇒ Compared to mild PI (0–2.5): mean = 99.56 °F ⇒ Compared to moderate PI (2.5–4): mean = 99.84 °F • Uncomfortable climate conditions linked to: ⇒ Higher mean heart rate ⇒ Higher breathing rate than under mild/moderate conditions
Smith et al. [37]	2020	South Georgia, USA	Cross-sectional	Migrant farmworkers	60	Occupational heat exposure	Descriptive statistics	• Over 50% incorrectly answered HRI first aid pilot questions • Common HRI symptoms: heavy sweating, muscle cramps • More than two-thirds experienced at least one HRI symptom during workday • Mean liquid consumption: 72.95 oz/day (less than recommended 32 oz/hour)
Mizelle et al. [47]	2022	North Carolina, USA	Mixed-methods-community-informed, sequential exploratory design	Latino male farmworkers	30	Occupational heat exposure	Content analysis of focus group discussions Descriptive statistics, correlations, *t*-tests and ANOVA	• Pre-work: 46.7% of farmworkers dehydrated • Post-work: 100% of farmworkers dehydrated • Work duration: 2 to 7.5 h in conditions above recommended heat exposure limits • Extreme heat exposure, inconsistent occupational policy compliance • Poor water quality and accessibility limited hydration • Findings indicate extreme heat, few work breaks, substandard housing
Mirabelli et al. [29]	2010	North Carolina, USA	Cross-sectional	Latino farmworkers from 52 migrant housing camps	300	Occupational heat exposure	Descriptive analysis, log binomial regression	• 94% reported working in extreme heat • 40% reported HRIs • Lower heat illness prevalence among H-2A workers
Kearney et al. [38]	2016	Eastern North Carolina, USA	Cross-sectional	Latino farmworkers from five migrant housing camps	158	Occupational heat exposure	Descriptive statistics—means and standard deviations chi-square tests or Fischer’s exact, logistic and log-binominal regression	• 72% experienced at least one HRI symptom • 27% had three or more HRI symptoms • Farmworkers lacked proper cooling methods • Sun Safety Behaviour • 85% wore long-sleeved shirts • 98% wore long pants • 93% wore baseball caps
Zhang et al. [48]	2016	Colorado, USA	Longitudinal	Patients visiting health centre in Colorado during the summer of 2013	14,481; including 150 migrant farmworkers and 231 seasonal farmworkers	Occupational heat exposure	Time series analysis, sensitivity analyses	• Higher clinic visits on hot days (88% increase; 95% CI, 26.2–180.0%) among migrant farmworkers
Li et al. [49]	2019	China	Cross-sectional	Ethnic minority groups in Pengshui Miao and Tujia Autonomous County of Chongqing	624	Hot weather	Descriptive statistics, multivariate logistic regression models	• Low willingness to see a doctor among ethnic minority residents (17.5%) • Higher perception of hot temperature among ethnic minority residents than Han residents • Lower perception of physical discomfort (*p* < 0.001) • Lower perception of aggravated diseases (*p* = 0.001) • Ethnic minority, outdoor working hours health status influencing factors for perceptions, subsequently for coping behaviours
Ye et al. [50]	2018	China	Cross-sectional	Ethnic minority groups in the Enshi Prefecture, primarily the Tujia and Miao ethnic groups	643	Hot weather	Descriptive, multivariate logistic regression models	• 61.3% perceived increasing temperatures • 73.2% viewed extreme heat as a health threat • 61.3% reported discomfort during hot weather • Only 12% had knowledge to stay healthy • 24.2% were prepared for extreme heat • Ethnic group, health status, employment influencing factors for perception
Sharpe and Wolkin [39]	2021	USA	Cross-sectional	Individuals who died as a result of natural disasters and extreme weather events in the USA between 1999 and 2018	N/A (analysis of population-level mortality data)	Various types of natural disasters and extreme weather events: excessive heat and cold, exposure to sunlight, lightning strikes, earthquakes, volcanic eruptions, avalanches, landslides, cataclysmic storms, floods and exposure to other unspecified forces of nature	Age-adjusted mortality rate calculations by race, ethnicity and hazard type; age-adjusted mortality rate ratios by race, ethnicity and state; geographic mapping of disparities in mortality by race and ethnicity across different states	• Mortality Rate ⇒ Non-Hispanic White: 0.38 per 100,000 ⇒ Non-Hispanic Black: 1.87 times higher (0.71 per 100,000) than non-Hispanic White ⇒ Non-Hispanic American Indian/Alaska Native: 7.34 times higher (2.79 per 100,000) than non-Hispanic White • Greatest causes of mortality—extreme heat and cold for all racial and ethnic groups
Albahsahli et al. [40]	2023 (Preprint)	California, USA	Mixed-methods—semi-structured interviews and geo-referenced data on climate-related disasters	Syrian and Iraqi refugees	67	Extreme heat, cold temperatures, dust storms and environmental conditions in refugee camps	Inductive thematic analysis Geo-referenced data on climate-related disasters	• Perception: Hypertension linked to stress from war, not extreme weather • Poorer mental health due to harsh weather conditions • Difficulty adjusting to different climates • Refugees vulnerable to climate-sensitive exposures, not fully aware of the health impacts
Méndez et al. [51]	2020	California, USA	Qualitative case study	Undocumented Latino/a and Indigenous immigrant communities	30	Thomas Fire	Qualitative analyses, including participant observations, interviews and thematic analysis of the gathered data	• Vulnerable to disasters, require special consideration in disaster planning • Disproportionately affected by racial discrimination, exploitation, economic hardships, less English and Spanish proficiency, and fear of deportation • Emergency response and recovery efforts ignored needs of the study population Resources directed to wealthy individuals
Ruttan et al. [41]	2013	USA	Retrospective cohort	Undocumented border crossers	N/A (analysis of population-level mortality data)	High temperature (Daily High Temperature or heat exposure?)	Descriptive statistics, Kruskal–Wallis equality-of-populations rank test, Logistic regression, Fractional polynomials, Hosmer–Lemeshow goodness-of-fit test, sensitivity analysis	• At Daily High Temperature of 40 °C, probability of at least one heat death—50% • Strong association between ambient temperature and heat death probability
Lane et al. [42]	2023	Atlanta, USA	Mixed methods	First-generation immigrants from Latin America	54	Climate change–related environmental and structural factors	Descriptive statistics, Kendall’s tau tests, grounded theory analysis	• Challenges: ran out of medication (daily medication users) (54%), food (37%), trouble paying utility bills (31%), no air conditioning (10%), language barriers, healthcare access, poor mental health • Emergency Preparedness ⇒ Knowledge of emergency alerts—65% ⇒ Resilience factor—social support
Taylor et al. [43]	2018	USAtates	Retrospective cohort	Individuals (non-US citizens compared to US citizens) who died from excessive heat exposure from January 1, 2005, through December 31, 2014	N/A (analysis of population-level mortality data)	Heat exposure	Descriptive statistics, Pearson’s chi-square test	• Heat-related deaths: ⇒ Non-US citizens: 2.23% (*n* = 999) ⇒ US citizens: 0.02% (*n* = 4196) • Age-adjusted standardised mortality ratio: ⇒ Non-US citizens vs. US citizens: 3.4 (95% CI = 3.2, 3.6) • Higher risk groups: ⇒ Hispanic non-US citizens: RR = 3.6 (95% CI = 3.2, 3.9) ⇒ Non-US citizens aged 18–24 years: RR = 20.6 (95% CI = 16.5, 25.7)
Tilstra et al. [44]	2022	Canada	Ecological correlational	Older adults and immigrants living in dissemination areas (DAs) in Edmonton	N/A (analysis of population-level health outcomes data)	Climate-related hazards: Annual average minimum, maximum and mean temperatures, diurnal temperature range, total yearly precipitation and the number of heat events Air pollution-related hazards: Ozone (O3), fine particulate matter (PM2.5), and nitrogen dioxide (NO2)	Descriptive statistics, negative-binomial regression to determine prevalence rate ratios (PRRs), main effects models	• Lower rates but increasing diurnal temperature range raised respiratory health risks in areas with more refugees (PRR 1.205 [95% CI 1.004, 1.447] at 20%) • Industrial Emission Effects • Increased injury rates: (PRR 1.127 [95% CI 1.058, 1.200] • Increased respiratory health rates (PRR 1.130 [95% CI 1.050, 1.216] at 20% refugee population • Similar effects on mental health event rates with higher total immigrants
Mercereau et al. [45]	2017	France	Ecological	Individuals who died in France between 2000 and 2009, both foreign-born and native-born	N/A (analysis of population-level mortality data)	Daily temperature (hot and cold)	Generalised additive model (GAM), estimating minimum mortality temperatures (MMTs), attributable fractions of deaths to cold and heat	• Temperature-mortality relationship and Minimum Mortality Temperature similar between foreign-born and natives • Attributable mortality to cold higher in foreign-born in some instances • Adaptation to the temperature patterns of continental France, even for those born in Africa
Uejio et al. [52]	2010	Philadelphia and Phoenix, USA	Ecological	Individuals living in neighbourhoods in Philadelphia, Pennsylvania, and Phoenix, Arizona	1741 census block groups in Philadelphia for the 1999 summer season and 1704 census block groups in Phoenix for the 2005 summer season	Extreme heat	Generalised linear mixed effect models (GLMM)	• Phoenix ⇒ Neighbourhoods with higher heat exposure—more heat distress calls ⇒ Higher proportions of Black, Hispanic, linguistically and socially isolated residents associated with more heat distress calls • Philadelphia ⇒ Neighbourhoods with low housing values: higher heat mortality ⇒ Neighbourhoods with higher Black residents: increased heat mortality
Voelkel et al. [53]	2018	City of Portland, Oregon, USA	Ecological	Residents (with different demographic characteristics) of the City of Portland, Oregon	640,000 residents of Portland, Oregon, as of 2016	Extreme heat, urban heat	Mapping of UHI Data, Student’s *t*-test, Hypothesis Testing, Network Distance Analysis, Covariance Analysis	• Groups in poverty and non-White populations at higher risk for heat exposure • Limited access to public cooling centres and residential central air conditioning • Significant relationships between heat exposure and socio-demographic factors such as income, race, education, age and English-speaking ability
Hansen et al. [46]	2014	Australia	Qualitative	Culturally and linguistically diverse (CALD) communities Respondents—migrants, refugees and stakeholders such as state and local government, non-government organisations and migrant and refugee health services	36	Extreme heat	Qualitative data analysis— Framework Analysis, Thematic Analysis, Synthesis, Deductive and Inductive Analysis	• Migrants and refugees generally adapt well but face sociocultural barriers • Barriers include socioeconomic disadvantage, poor housing, language barriers, isolation, health issues, cultural factors and lack of acclimatisation • High-risk groups—new arrivals, people in new and emerging communities, older migrants

The overall quality of the included studies ranged from high (*n* = 23) to moderate (*n* = 2). One cross-sectional study did not provide sufficient information to confirm the rigour of its data collection and analysis processes [29]. Similarly, one mixed-method study lacked adequate detail to assess the robustness of its data analysis [30]. A detailed quality appraisal is provided in Supplementary File 2.

The following sections present the three key themes identified in the review: health impacts of climate change and related weather events; environmental and sociocultural factors; and adaptive strategies and barriers.

### Theme 1: Health Impacts of Climate Change and Related Weather Events Among CALD communities

Climate change and related weather events have profound and multifaceted impacts on the physical and mental health and wellbeing of CALD communities. Three-quarters of the studies (*n* = 19) reported that such events adversely affected health outcomes in these communities to some extent [29–47], while the remaining six studies partially addressed these impacts [48–53]. Reported physical health impacts included heat-related illnesses, exacerbation of existing chronic diseases, respiratory issues, dehydration, injuries and deaths, while mental health impacts encompassed post-traumatic stress disorder (PTSD), depressive symptoms and anxiety.

#### A. Mental Health Impacts

Six studies from various regions of the USA examined the mental health effects of disasters such as Hurricane Maria, Hurricane Harvey and Hurricane Katrina [31–36]. These impacts were not only linked to the disasters themselves but also to post-disaster experiences like cultural stress, social support, health services access and language barriers [31–36]. Across all studies, mental health symptoms were prevalent among hurricane survivors. For instance, the prevalence of five or more depressive symptoms increased significantly from 34% at baseline to 49% 1 year post-Katrina among Vietnamese immigrants [31]. Similarly, studies among Vietnamese Americans post-Hurricane Harvey highlighted poorer mental health and a high prevalence of depressive symptoms, with over half of the participants experiencing five or more symptoms [33, 34]. In study on Hurricane Maria survivors, 20.5% reported PTSD scores of 50 or higher, suggesting a probable PTSD diagnosis persisting 3 to 4 years post-disaster [36].

Several risk factors were linked to poor mental health outcomes, including physical injury, property loss, social isolation, lower income and cultural stressors such as discrimination, language barriers and poor acculturation [31, 33–36]. For example, immigrants who sustained serious injuries during Hurricane Harvey were three times more likely to report poor mental health compared to those without such injuries (OR = 3.34, 95% CI 1.12–9.94, *p* = 0.03) [33]. Conversely, protective factors included social support, post-disaster health care access, higher English language proficiency, low levels of cultural stress and higher household income [33–35].

Other than hurricane studies, a mixed methods study in San Diego found that poor weather conditions and difficulty adjusting to new climates contributed to mental distress among resettled Syrian and Iraqi refugees [39].

#### B. Physical Health Impacts

##### Heat-Related Illness Symptoms

Six studies conducted among migrant farmworkers, including Hispanic and Latino workers across various regions of the USA such as North Carolina, South Georgia, the Midwest and Colorado, primarily reported on occupational heat-related illness (HRI) symptoms [29, 30, 37, 38, 47, 48]. Some also examined hydration and related physiological parameters [30, 37, 47]. Reported HRI symptoms included fainting, muscle cramps, headaches, heavy sweating, weakness, fatigue, dizziness, light-headedness, rash, nausea, vomiting, confusion, extreme thirst/dehydration and stomach cramps [29, 30, 37, 38].

For instance, nearly 72% of Latino farmworkers in North Carolina reported experiencing at least one HRI symptom during hot months (mean maximum temperatures, 30–35 °C), with headaches (43.6%), heavy sweating (37.6%) and muscle cramps or spasms (35.7%) being most common symptoms [38]. In South Georgia, the most frequently reported HRI symptoms were heavy sweating (50%) and muscle cramps (25%) [37].

On the other hand, farmworkers with H-2A visas (temporary agricultural work permits) reported lower rates of HRI symptoms (31%) compared to those without such visas (56%) [29]. A qualitative study from Australia which did not exclusively focus on farmworkers reported other heat-related illness symptoms such as allergies, feeling lazy, itchy skin, kidney problems, nutritional deficiencies, gallstones, constipation and sunburn among migrants due to heat exposure [46].

##### Chronic Disease and Related Risk Factors

Chronic conditions were found to increase vulnerability to climate-related health issues; however, few studies focused on the impacts of climatic events on chronic diseases and related risk factors [30, 40, 44, 46, 48]. A mixed methods study in San Diego among Syrian and Iraqi refugees reported asthma related to dust storms, as well as illnesses linked to contaminated water [40]. An ecological study in Edmonton, Canada, found that areas with refugees were more vulnerable to respiratory issues due to changes in temperature and air pollution [44].

In China, two cross-sectional studies found that 60–90% of ethnic minority participants reported feeling physical discomforts during hot weather conditions [49, 50]. These, along with an Australian qualitative study, reported that participants perceived hot weather exacerbated pre-existing chronic health conditions and increased their vulnerability to heat [46, 49, 50]. However, clinic visits due to heat-related illnesses were generally low, despite high perceptions of risk. In China, only 24.4% of the Han population were willing to visit a doctor for physical discomfort caused by hot weather, with even lower willingness among ethnic minority residents (17.5%) [49]. Similarly, only 2.7% of Hispanic farmworkers in the USA visited an on-site medic clinic for dehydration and heat-related symptoms [30]. Contrastingly, a Colorado showed that higher temperatures led to an average increase of 88% in daily clinic visits among migrant farmworkers, although no significant link to cardiovascular-specific outcomes was found [48]. A study in Australia highlighted that past experiences and lack of culturally appropriate care may prevent migrants from seeking necessary medical attention [46].

Two studies reported on chronic disease risk factors such as obesity and hypertension [30, 40]. A study among Hispanic farmworkers in the USA found that obesity significantly affected heat-related symptoms, with obese (BMI 31–39) and morbidly obese (BMI ≥ 40) workers more likely to report extreme thirst, muscle cramps and dizziness [30]. Additionally, younger participants showed higher rates of overweight and exhibited more heat stress symptoms compared to older workers [30]. While dust exposure was reported to aggravate hypertension among Syrian and Iraqi refugees in San Diego, no direct link between extreme weather and hypertension was found [40].

##### **Hydration Issues**

Fluid intake and hydration status among CALD groups were examined in four studies, primarily involving migrant farmworkers [30, 37, 46, 47]. Farmworkers mostly consumed water but also relied on soft drinks, soda, sports drinks, sugar-sweetened and caffeinated drinks, and in one study, beer and other forms of alcohol [30, 37, 46, 47].

Most studies indicated that farmworkers and immigrants were not hydrating adequately. In South Georgia, migrant farmworkers consumed only 72.95 oz of water per day, which is significantly less than the recommended 32 oz of water per hour for high heat labour [37]. Similarly, in North Carolina, none of the farmworkers were significantly dehydrated pre-shift, but all were dehydrated post-shift, with 8% being severely dehydrated [47].

Several studies reported a reliance on sugary or caffeinated drinks. For instance, 23.23% South Georgia migrant farmworkers consumed sports drinks, 11.47% soft drinks, and 3.66% energy drinks during work [37]. Similarly, a study in the Midwestern United States found the majority (68.9%) of Hispanic farmworkers drank water, though many also consumed beverages high in sugar and caffeine [30].

A qualitative study from Australia reported other health consequences such as incontinence, particularly among older immigrants, dehydration, kidney stones and tooth pain from cold water [46]. Socio-cultural factors influencing fluid intake are discussed further in Theme 2.

##### Injuries and Mortality

Seven studies reported on injuries and mortality due to various weather events among CALD groups [33, 34, 39, 43–45, 52]:

**Injuries:** Two studies conducted among Harvey survivors reported that over a quarter sustained hurricane-related injuries [33, 34]. As reported, those with serious injuries had significantly higher odds of poor physical health (OR = 3.19; 95% CI, 1.33–7.65; *P* = 0.01) compared to those without serious injuries or illnesses [33]. A study conducted in Canada reported that higher exposure to industrial pollution resulted in more injuries, particularly in areas with higher populations of immigrants and refugees [44].

**Mortality:** Several studies highlighted disparities in mortality rates due to extreme weather events, particularly among racial and ethnic groups [39, 43, 52]. A study from the USA found that non-Hispanic American Indian/Alaska Native (AI/AN) people had the highest heat and cold-related mortality rate (2.79 per 100,000), followed by Non-Hispanic Black people (0.71), both significantly higher than non-Hispanic White people [39].

A study among undocumented the USA border crossers reported that 55% of 1457 deaths were attributed to heat-related causes, making heat exposure the leading cause of mortality in this group [41]. Another US study found that non-citizens had higher proportions of heat-related deaths (2.23%) compared to the US citizens (0.02%), with 19.8% of non-US citizen deaths occurring on farms [43]. Additional risk factors for heat-related deaths among non-US citizens were found to be younger age and Hispanic ethnicity [43].

An ecological study conducted in the USA found that neighbourhoods with a higher proportion of Black residents in Philadelphia were more likely to report heat-related mortality [52]. Similarly, in Phoenix, neighbourhoods with higher populations of Black, Hispanic, linguistically and socially isolated residents made more heat distress calls, highlighting racial disparities in heat-related health outcomes [52]. Conversely, a study from France found no difference in temperature-mortality between foreign-born and native-born populations, suggesting effective climate adaptation across groups [45].

### Theme 2: Environmental and Socio-cultural Determinants of Health

The health and wellbeing of CALD communities are strongly influenced by various environmental and socio-cultural factors. These factors shape not only their baseline health status but also their vulnerability to extreme weather events and climate change, often exacerbating health disparities.

Several studies underscored the importance of strong social support networks as a key protective factor. Immigrants embedded in cohesive communities with robust community connections were more likely to secure stable housing, find jobs and access essential resources, thereby improving their ability to cope with climate related stressors [42].

Access to culturally appropriate and affordable healthcare emerged as another pivotal determinant in mitigating health impacts during and after extreme weather events [33, 42, 46, 51]. For example, a study conducted in Texas revealed that only 16.8% of Vietnamese American residents received free healthcare after Hurricane Harvey. Importantly, this access to free healthcare was significantly associated with better mental health [33]. Additionally, the same study reported that health insurance coverage may have protected against poor physical health outcomes [33]. However, another study noted that despite 65.83% of surveyed Hurricane Harvey survivors having health insurance, insurance status did not significantly associate with depressive symptoms, suggesting access alone may not be sufficient if other barriers persist [34]. Structural barriers limit healthcare for certain groups. A study conducted in the midwestern region of the United States revealed that many farmworkers did not obtain timely medical assistance for HRI symptoms, despite the availability on-site clinics [30]. Key barriers reported were lack of health insurance, fear of deportation and lack of knowledge on navigating the healthcare system [33, 42, 51].

Similarly, a study focusing on undocumented CALD populations in the USA reported that these communities were disproportionately affected by the Thomas Wildfire due to pre-existing vulnerabilities such as low socioeconomic status, uncertain legal status and inadequate or lack of access to healthcare resources [51].

Poor living conditions were frequently cited as environmental risk factors that increase vulnerability to climate-related health threats. Multiple studies reported that migrant farmworkers and other CALD groups often live in substandard housing lacking adequate cooling facilities like air conditioners [42, 46, 48]. Mobile homes or temporary housing units, specifically common among farmworkers, offered little protection against heatwaves and other weather extremes [48]. Housing-related issues such as mould, pest infestations, water leaks and lack of air conditioning were reported [42, 46]. A mixed method study of first-generation immigrants in metropolitan Atlanta found that 46% reported at least one issue in their house, including the damage from storms, floods and fires [42]. Economic constraints further compound these issues. An Australian study revealed that high electricity costs often forced low-income migrants to make difficult choices between comfort and financial stability, increasing health risks during heatwaves [46].

Limited social mobility and transportation access also contribute to heightened climate vulnerability. A few studies reported that CALD groups faced difficulties in reaching cooling centres or other safe locations during extreme heat [46, 53]. Social isolation, especially among elderly migrants without close family support or community networks, was reported to worsen health risks during emergencies [46]. Conversely, strong social networks and familial support among CALD groups were reported as protective factors especially during and after environmental crises, and particularly among older CALD individuals [34–36, 46]. For instance, Vietnamese Americans with strong community ties reported better mental health outcomes post-Hurricane Harvey, even when accounting for hurricane-related injuries and damages [34].

Acculturation was also identified as a protective factor. Increased levels of acculturation protected against poor mental health outcomes among Vietnamese American Hurricane Harvey survivors, highlighting the potential benefits of cultural adaptation in enhancing coping capacities [33, 34].

In contrast, cultural stressors, such as discrimination and language difficulties, were reported to exacerbate health issues. Studies among Puerto Rican migrants found that experiences of discrimination and language-related stress contributed to poor mental health outcomes [32][35, 36]. Inadequate or lack of English language proficiency, in particular, was shown to hinder access to timely emergency health information, services and relief aid measures [35, 36, 46, 51]. On the other hand, Vietnamese Americans with better English skills were more likely to access recovery resources and reported less distress post-hurricane [33, 34].

### Theme 3: Adaptive Strategies and Barriers

Eight studies provided partial insights into the various adaptive and coping strategies employed by CALD communities to protect themselves against extreme weather events, along with the barriers they face in adapting and coping with the impacts of climate change [29, 30, 37, 38, 46, 47, 49, 50].

Four studies focused on migrant farmworkers reported their occupational adaptations to extreme heat. These workers employed various strategies to mitigate heat exposure such consuming various fluids to stay hydrated, taking breaks, resting in shaded areas like under trees and using air-conditioned spaces [29, 30, 37, 47]. For instance, in a North Carolina-based study among Latino farmworkers, 98% reported drinking more water, and 81% took rest breaks in shaded areas [29]. However, only 2% reported going to an air-conditioned place to rest during or after work [29].

Outside of occupational settings, studies conducted among ethnic minorities in China found high self-reported levels of adaptation to hot weather. Specifically, 98.4% of them reported taking adaptive actions such as drinking more water, staying indoors and using fans or air conditioners [49, 50]. In addition, a higher perception of warm temperatures was linked to more positive adaptive behaviours, such as seeking medical help or asking for assistance [49, 50]. Nevertheless, these studies also revealed lower awareness compared to the general population about the health risks posed by extreme heat, including the potential for disease aggravation.

CALD communities faced multiple barriers to adapting and coping with extreme weather events, including economic constraints, legal and documentation issues, language and literacy barriers, cultural practices, social isolation, poor housing conditions and limited access to healthcare [32, 37, 38, 46, 51]. For instance, high power costs restricted the use of air conditioning among low-income migrants, while undocumented immigrants avoided seeking medical help due to fear of deportation, exacerbating their vulnerability during extreme weather events [42, 46, 51]. A study conducted among undocumented immigrants in the USA reported that they experienced discrimination based on language and culture, and often lacked basic occupational health and safety information, further compromising their ability to adapt to climate change impacts [51].

In the Australian context, migrants and refugees faced a variety of sociocultural barriers that hindered their adaptive capacity to extreme heat. These included socioeconomic disadvantage, substandard housing, language obstacles, isolation, pre-existing health problems, cultural factors and lack of acclimatisation [46]. In addition, the study reported that many new arrivals may be unaware of the need to dress lightly during heat, while cultural and religious practices sometimes dictated wearing heavy, dark garments unsuitable for hot weather [46]. On the other hand, some immigrants often adapted culturally by using items such as burqini swimwear and sun-protective umbrellas. These adaptations allowed them to align with local lifestyles while maintaining their cultural values [46].

## Discussion

This review identified, synthesised and discussed the health and wellbeing impacts of climate change and related weather events on culturally, linguistically, ethnically and racially diverse communities. It included studies with varied study designs, focusing on a broad range of CALD groups, such as migrants, undocumented border crossers, seasonal agricultural workers and ethnic and racially diverse groups. The observed climate change-related health impacts ranged from physical health issues, such as heat-related illnesses, exacerbation of chronic diseases, respiratory problems, dehydration, injuries and increased mortality, to mental health issues such as PTSD, depressive symptoms and anxiety. The factors contributing to these negative health outcomes included limited culturally appropriate healthcare service availability and accessibility, poor housing and living conditions, inadequate social support and networks, occupational hazards, cultural stressors such as language challenges, discrimination and legal insecurities [33, 34, 42, 46, 48, 51]. Evidence demonstrates that CALD groups are disproportionately affected by climate-related health risks [5, 6, 39, 43, 53, 54]. For instance, the mental health impacts observed among hurricane survivors in this review align with global evidence on climate-related disasters exacerbating psychological distress and burden in immigrant populations [55].

Climate-sensitive health outcomes among CALD communities can be attributed to several interconnected factors. For instance, many CALD groups in the USA live in environments more vulnerable to the impacts of climate change, such as regions prone to intense heat waves, hurricanes, flooding and poor air quality [56]. These environmental conditions exacerbate existing health vulnerabilities, leading to an increased likelihood of climate-sensitive health outcomes. The climate change-related negative physical and mental health outcomes observed among CALD groups can in part be explained by lack of access to timely, culturally appropriate healthcare, which was particularly evident among some migrant groups living and working in the USA [57, 58]. As these migrant groups were predominantly seasonal agricultural workers, their relatively low income, lack of health insurance and insecure working conditions may prevent them from accessing timely healthcare, thereby contributing to poorer health outcomes in both the short and long term [59]. Access to culturally appropriate and affordable healthcare is pivotal for mitigating the health impacts of extreme weather events [42, 51, 60].

Similarly, limited English language proficiency contributed to poorer climate change-related health outcomes among CALD groups such as immigrants, migrants affected by hurricane disasters and undocumented migrants [32, 36, 51]. Language barriers have been linked to lower health literacy, difficulty understanding alerts, warnings, public health information and lower utilisation of healthcare services—particularly in communities where interpreter services are unavailable or difficult to access [32, 36, 51, 61–63]. To address this barrier, it is critical for governments, emergency services and healthcare service providers to adopt inclusive, multilingual risk communication strategies that enhance clear understanding across diverse linguistic groups [64, 65]. For instance, a case study conducted in one region of Australia showed that bilingual CALD community members effectively interpreted emergency notifications through a citizen translation approach called as Collaborative Translation of Emergency Messages (Co-TEM). This approach included strategies such as simplifying official messages, providing translator training for bilingual members, utilising technologies such as online dictionaries and mobile apps and engaging communities in emergency preparedness and response efforts [66]. Such community-focused strategies may also strengthen community networks and enhance social support among CALD communities, which are widely recognised as crucial factors for reducing negative mental health outcomes associated with climate change–related extreme weather events [67–69].

Proper hydration is essential to mitigate the negative health impacts of heat exposure; however, several barriers to maintaining adequate hydration were identified among CALD groups, particularly migrant farmworkers in the USA [30, 37, 46, 47]. These barriers included limited or difficult access to drinking water, poor water quality, level of work intensity, workplace conditions and inadequate awareness of proper hydration measures [30, 47, 70–72]. Migrant farmworkers may face pressure to work long hours without taking rest breaks, and many may fear losing work hours, shifts and their job if they take breaks [73]. Migrant farmworkers who are undocumented or on temporary visas face increased difficulties due to their legal status and fear of deportation [72]. Thus, it is critically important for workplaces and employers to ensure that workers have easy access to clean drinking water and specialised rehydration drinks or powders to support their hydration requirements, particularly during periods of extreme heat [74].

Some studies reported that migrant farmworkers rehydrated by consuming sweetened, caffeinated and alcoholic beverages, which can exacerbate dehydration and increase the risk of conditions such as renal dysfunction, kidney stones and chronic kidney disease [75–79]. These drinks are often consumed to boost work productivity and alertness, and alleviate body pain and discomfort, and because they are more readily available and accessible [70, 80, 81]. Furthermore, taste preferences, social desirability, inadequate awareness of rehydration guidance and past experiences with low-quality drinking water were some of the additional reasons why CALD group members were hesitant to drink sufficient water to maintain adequate hydration [46]. This indicates that hydration practices are influenced by cultural norms, preferences and experiences, which should inform the development and design of targeted community health education and guidance on rehydration strategies [82].

CALD groups, such as low-income immigrants and migrant farmworkers, often live in substandard conditions such as housing with no or inadequate cooling facilities, mould, pest infestations and water leaks, which increases their susceptibility and vulnerability to the negative impacts of climate change–related extreme weather events including heat stress and infectious diseases, such as malaria and dengue fever [42, 46, 73]. This is especially problematic in mobile homes or temporary housing units, which are particularly common among seasonal farmworkers [83–86]. Farmworker camps in the USA have been found to have dangerously high heat indices in most rooms, even in those with air conditioning [84, 85]. Economic constraints further exacerbate these problems, making it difficult for many low-income migrants to justify spending on home repairs and improvements, which in turn increases health risks during heatwaves and other climate change–related extreme weather events [6, 46, 87].

The observed higher mortality rates due to extreme heat among racial and ethnic minority groups and undocumented border crossers can in part be attributed to greater social vulnerability [3, 88, 89], which is associated with factors that increase heat exposure, such as living and working in environments with limited green space and more heat-absorbing surfaces, and the lack of structural adaptations like air conditioning and adequate ventilation [89, 90]. There is an urgent need for increased focus on identifying urban high heat areas, also known as ‘heat islands’, and implementing evidence-based mitigation strategies such as updating building codes, and implementing green infrastructure projects to reduce the risk of heat-related morbidity and mortality [91]. Heat adaptation for human health is an unexplored area that merits further research attention in the context of the increasing negative health impacts of climate change [92, 93]. Such evidence is critical to inform the development of public health policies and programs that focus on reducing identified vulnerabilities and inequities among CALD communities [94].

While this review focused on the health impacts of climate change and related weather events, it is important to note that many of the observed health outcomes are not solely attributable to these events. Socio-economic status (SES) plays a critical mediating factor. Individuals and communities with lower SES often face multiple intersecting vulnerabilities such as low income, poor housing, precarious employment and reduced access to healthcare and social services, which compound the health risks associated with climate change [55, 95]. These socioeconomic disadvantages may increase exposure to environmental hazards, while simultaneously reducing adaptive capacity [96]. So, it can be said that the relationship between climate change and health in CALD communities is shaped not only by environmental factors but also by broader structural inequities.

High levels of risk perception were found to substantially increase the adoption of coping strategies and behaviours among ethnic minorities in China [49, 50]. While the reasons for some CALD community members having a higher risk perception of heat in comparison to the general population are not entirely clear, it has been suggested that traditional and cultural ways of living may contribute to greater connection and sensitivity to changes in the environment and climate [50, 97]. An enhanced understanding of risk perception and its link to adaptive behaviours is important for developing and implementing targeted policies, public health interventions and heat warning systems to encourage adaptive behaviours [98]. Future research and design of educational materials and resources should consider tailoring culturally and linguistically appropriate health education utilising multiple channels to reach CALD communities such as community centres, social media and religious institutions, and through the active engagement of community leaders.

### Implications for Policy and Practice

More than three-fourths of reviewed studies provided comprehensive recommendations to improve the adaptation and coping strategies of CALD groups facing climate change impacts and extreme weather events. These recommendations mainly highlighted the importance of targeted interventions, policy reforms and community-based approaches to reduce the adverse effects of climate change and extreme weather on these vulnerable populations [99].

Findings from this review suggest that it is crucial to develop culturally relevant resources and risk communication strategies that are easily accessible in multiple languages, thus enabling CALD communities to receive timely, clear and comprehendible information to guide decision-making. Providing effective and reachable education and warning messages to CALD groups through approaches based on local knowledge and proven practices such as using a variety of communication methods, ensuring messages are clear and specific, and creating coordinated campaigns that involve CALD communities in planning and implementation is essential to success [54, 100]. Additionally, enhanced occupational health and safety measures are imperative for protecting vulnerable workers, particularly migrant farmworkers. Workplaces have a responsibility to implement strategies to protect workers’ health and wellbeing, such as facilitating adequate hydration, mandating rest breaks, monitoring environmental conditions and adapting work-rest ratios. Employers need to be educated on the importance of these measures and supported in implementing them effectively. Ensuring the health and safety of farmworkers is crucial, given their significant exposure to extreme heat and other climate-related risks [101].

There is a vital need for multilingual health and support services and targeted mental health interventions to effectively address the needs of diverse communities during and after climate change-related extreme weather events. Culturally relevant physical and mental health services can mitigate psychological distress and support recovery. Studies have shown that social support networks and community cohesion significantly contribute to mental wellbeing, emphasising the need for programs that develop, strengthen and build upon existing social networks within CALD communities [54].

Substandard housing conditions substantially increase the vulnerability of CALD communities to climate change-related health issues. Policies and guidelines should prioritise housing and infrastructure improvements to ensure adequate cooling, ventilation and structural integrity, especially among socioeconomically disadvantaged and marginalised CALD communities. Economic constraints, such as high utility costs, exacerbate health risks during extreme weather events, highlighting the need for financial assistance programs. Addressing these housing and economic issues can significantly reduce the health risks faced by these communities [102].

### Knowledge Gaps and Future Research Directions

The review identified some knowledge gaps such as understanding how CALD groups manage health and wellbeing impacts from climate change and extreme weather events in host countries. Also, there is limited information on effectiveness of different climate change adaptation strategies adopted by CALD groups and how their cultural backgrounds influence their capacity to cope. Most of the reviewed studies used small sample sizes limiting the generalisability of findings. Future research should include larger, more diverse samples to better represent various CALD groups and their unique vulnerabilities. Likewise, more longitudinal research is needed to track the long-term health outcomes and effectiveness of adaptation approaches of CALD groups affected by climate change [103]. Importantly, future studies should focus on underexplored regions such as low- and middle-income countries in the Global South, assessing the vulnerability of CALD communities to climate change health impacts [104]. The impacts of climate change in the Global South can be more severe and widespread, with fewer available resources, infrastructure and policies to adequately respond to the associated health challenges. Understanding regional differences in vulnerability can inform more tailored and effective interventions, ensuring the unique needs of each community are met.

### Strengths and Limitations of the Review

This review had several strengths and limitations. One major strength is that, to our knowledge, this is the first comprehensive synthesis of evidence in this field. The review utilised a systematic search strategy and a rigorous approach to assessing the reliability and quality of the included studies. In addition, the inclusion of diverse study designs enabled a holistic overview of the existing research in this field. This review had some limitations. First, only English language publications were included due to the lack of access to reliable translation resources. While this was a necessary constraint, it may have resulted in the exclusion of relevant studies published in other languages, potentially limiting the representation of certain countries or regions. Second, the review was limited to studies published from 2010 onwards which may have led to the omission of earlier but still relevant research. Third, it is important to acknowledge that given the diversity of CALD communities worldwide, the review findings may not be universally applicable or fully representative of the broader CALD population. Additionally, most of the included studies were conducted in the USA, which impacts the generalisability of the findings. Therefore, the findings should be interpreted with caution, particularly in relation to CALD communities in lower income and non-Western contexts.

## Conclusion

This review synthesised available global evidence on the health impacts and vulnerabilities faced by culturally and linguistically diverse (CALD) communities in the context of climate change and related weather events. The findings showed that these communities face heightened physical and mental health risks, further compounded by poor housing conditions, limited access to culturally appropriate healthcare, cultural stressors and inadequate sensitisation to climate-related health impacts. To address these issues, governments and policymakers must prioritise the integration of cultural and linguistic considerations into all levels of climate and health policy, ranging from emergency preparedness to long-term urban planning and housing improvements. Likewise, policies should require employers to ensure safe and equitable working conditions for migrant workers, underpinned by culturally and linguistically appropriate occupational health and safety measures. Finally, more longitudinal and contextually diverse research, particularly in non-Western settings, is needed to better understand the evolving health experiences and needs of CALD population in a changing climate.

## Supplementary Information

Below is the link to the electronic supplementary material.

ESM 1 (DOCX 14.7 KB)

ESM 2 (DOCX 25.8 KB)

## Data Availability

Not applicable.
