# Supplementary material for: Impacts of Climate Change and Related Weather Events on the Health and Wellbeing of Culturally and Linguistically Diverse Communities: A Systematic Review
Source: J Racial Ethn Health Disparities. 2025 Jun 26;13(5):3673–700. doi: 10.1007/s40615-025-02527-1 (PMC13546328; doi:10.1007/s40615-025-02527-1)
Supplement: Supplementary file 1 [file 40615_2025_2527_MOESM1_ESM.docx]

**Health and Wellbeing Impacts of Climate Change among Culturally and Linguistically Diverse Communities: A Systematic Narrative Synthesis**

**Supplementary File 1**

## Search queries and yields (January 2010 – March 2024)

PubMed Search Query**:** This search yielded 297 articles in total.

(("Transients and Migrants"[mh] OR "Cultural diversity"[mh] OR "Ethnic and racial minorities"[mh] OR "Emigrants and Immigrants"[mh] OR "refugees"[mh] OR "Culturally and linguistically diverse"[tiab] OR CALD[tiab] OR Ethnic minorit*[tiab] OR Migrants[tiab] OR Immigrants[tiab] OR Asylum seeker*[tiab] OR Refugee*[tiab] OR Newcomers[tiab] OR Diverse cultural background[tiab] OR Multi-cultural background[tiab] OR "Transients and Migrants"[tiab] OR Cultural diversity[tiab] OR "Ethnic and racial minorities"[tiab] OR "Emigrants and Immigrants"[tiab]) AND ("Climate change"[mh] OR "extreme heat"[mh] OR "Hot temperature"[mh] OR "Wildfires"[mh] OR "Droughts"[mh] OR "Cyclonic storms"[mh] OR "Floods"[mh] OR Climate change[tiab] OR extreme heat[tiab] OR hot weather[tiab] OR Hot temperature[tiab] OR Wildfire*[tiab] OR Droughts[tiab] OR Cyclonic storm*[tiab] OR Flood*[tiab] OR heat wave*[tiab] OR bushfire*[tiab] OR cyclone*[tiab] OR hurricane*[tiab] OR storms[tiab])) AND ("Health"[mh] OR "Health status"[mh] OR "morbidity"[mh] OR "mortality"[mh] OR "heat stroke"[mh] OR "cardiovascular diseases"[mh] OR "kidney diseases"[mh] OR "dehydration"[mh] OR "Heat stress disorders"[mh] OR "respiratory tract diseases"[mh] OR "Mental health"[mh] OR Health[tiab] OR Health status[tiab] OR morbidit*[tiab] OR mortality[tiab] OR heat stroke[tiab] OR cardiovascular diseases[tiab] OR kidney diseases[tiab] OR dehydration[tiab] OR Heat stress disorders[tiab] OR respiratory tract diseases[tiab] OR Mental health[tiab] OR hospital admission*[tiab] OR heat injur*[tiab] OR Well being[tiab] OR wellbeing[tiab] OR Heat-related illnesses[tiab] OR Heat Stress[tiab] OR Heat stroke[tiab] OR Respiratory disease*[tiab] OR Renal disease[tiab] OR Dehydration[tiab] OR Heat rash[tiab])

Scopus Search Query**:** This search yielded 35 articles in total.

( TITLE ( "Culturally linguistically diverse" OR "CALD" OR "Ethnic minorities" OR "Migrant*" OR "Immigrant*" OR "Asylum seekers" OR "Refugee*" OR "Diverse cultural groups" OR "Multicultural background" ) AND TITLE ( "Climate change" OR "Climate change events" OR "Extreme heat" OR "Heat wave*" OR "Hot weather" OR "Bushfires" OR "Drought*" OR "Cyclone*" OR "Hurricane*" OR "Flood*" OR "Storms" ) AND TITLE-ABS-KEY ( "Health" OR "Wellbeing" OR "Heat-related illnesses" OR "Heat Stress" OR "Heat stroke" OR "Mortality" OR "Morbidity" OR "cardiovascular diseases" OR "Respiratory diseases" OR "Mental Health" OR "Renal disease" OR "Health status" OR "Dehydration" OR "Heat rash" OR "Heat injur*" ) )

Web of Science Search Query**:** This search yielded 14 articles in total.

((TI=("Culturally linguistically diverse" OR "CALD" OR "Ethnic minorities" OR "Migrant*" OR "Immigrant*" OR "Asylum seekers" OR "Refugee*" OR "Diverse cultural groups" OR "Multicultural background")) AND TI=("Climate change" OR "Climate change events" OR "Extreme heat" OR "Heat wave*" OR "Hot weather" OR "Bushfires" OR "Drought*" OR "Cyclone*" OR "Hurricane*" OR "Flood*" OR "Storms")) AND AB=("Health" OR "Wellbeing" OR "Heat-related illnesses" OR "Heat Stress" OR "Heat stroke" OR "Mortality" OR "Morbidity" OR "cardiovascular diseases" OR "Respiratory diseases" OR "Mental Health" OR "Renal disease" OR "Health status" OR "Dehydration" OR "Heat rash" OR “Heat injur*”)
