# Supplementary material for: Impacts of Climate Change and Related Weather Events on the Health and Wellbeing of Culturally and Linguistically Diverse Communities: A Systematic Review
Source: J Racial Ethn Health Disparities. 2025 Jun 26;13(5):3673–700. doi: 10.1007/s40615-025-02527-1 (PMC13546328; doi:10.1007/s40615-025-02527-1)
Supplement: Supplementary file 2 [file 40615_2025_2527_MOESM2_ESM.docx]

**Health and Wellbeing Impacts of Climate Change among Culturally and Linguistically Diverse Communities: A Systematic Narrative Synthesis**

**Supplementary File 2**

Table 3: Quality assessment of the included studies using modified CASP checklist

|  | Section A - Are the results valid? | | | | Section B - What are the results? | | | Section C - Will the results help locally? | | |
| --- | --- | --- | --- | --- | --- | --- | --- | --- | --- | --- |
| Author | Was there a clear statement of the aims of the research? Did the study address a clearly focused issue? | Was the research methodology appropriate to address the aims of the research? | Was the recruitment strategy appropriate to the aims of the research? Were the study participants (cohort/cases/control) recruited in an acceptable way? | Was the data collection appropriately rigorous? | Have ethical issues been taken into consideration? | Was the data analysis sufficiently rigorous? | Is there a clear statement of findings? How precise are the results? Do you believe the results? | Did the research identify research gap? Did the research contribute the body of knowledge? | Do the results of this study fit with other available evidence? | Were implications of study for policy and practice stated or mentioned? |
| Vu & VanLandingham[31] | Yes | Yes | Yes | Yes | Yes | Yes | Yes | Yes | Yes | Yes |
| Carl et.al[32] | Yes | Yes | Yes | Yes | Yes | Yes | Yes | Yes | Yes | Yes |
| Pham et.al[33] | Yes | Yes | Yes | Yes | Yes | Yes | Yes | Yes | Yes | Yes |
| Pham et.al[34] | Yes | Yes | Yes | Yes | Yes | Yes | Yes | Yes | Yes | Yes |
| Montero-Zamora et.al[35] | Yes | Yes | Yes | Yes | Yes | Yes | Yes | Yes | Yes | Yes |
| Hodges et.al[36] | Yes | Yes | Yes | Yes | Yes | Yes | Yes | Yes | Yes | Yes |
| Culp&Tonelli[30] | Yes | Yes | Yes | Yes | Yes | Not entirely | Yes | Yes | Yes | Yes |
| Smith et.al[37] | Yes | Yes | Yes | Yes | Yes | Yes | Yes | Yes | Yes | Yes |
| Mizelle et.al[47] | Yes | Yes | Yes | Yes | Yes | Yes | Yes | Yes | Yes |  |
| Mirabelli et.al[29] | Yes | Yes | Yes | Not entirely | Yes | Not entirely | Yes | Yes | Yes | Yes |
| Kearney et.al[38] | Yes | Yes | Yes | Yes | Yes | Yes | Yes | Yes | Yes | Yes |
| Zhang et.al[48] | Yes | Yes | Yes | Yes | Yes | Yes | Yes | Yes | Yes | Yes |
| Li et.al[49] | Yes | Yes | Yes | Yes | Yes | Yes | Yes | Yes | Yes | Yes |
| Ye et.al[50] | Yes | Yes | Yes | Yes | Yes | Yes | Yes | Yes | Yes | Yes |
| Sharpe & Wolkin[39] | Yes | Yes | NA (Not applicable) |  | NA | Yes | Yes | Yes | Yes | Yes |
| Albahsahli et.al[40] | Yes | Yes | Yes | Yes | Yes | Yes | Yes | Yes | Yes | Yes |
| Méndez et.al[51] | Yes | Yes | Yes | Yes | Yes | Yes | Yes | Yes | Yes | Yes |
| Ruttan et.al[41] | Yes | Yes | NA | Yes | NA | Yes | Yes | Yes | Yes | Yes |
| Lane et.al[42] | Yes | Yes | Yes | Yes | Yes | Yes | Yes | Yes | Yes | Yes |
| Taylor et.al[43] | Yes | Yes | Yes | Yes | NA | Yes | Yes | Yes | Yes | Yes |
| Tilstra et.al[44] | Yes | Yes | Yes | Yes | Yes | Yes | Yes | Yes | Yes | Yes |
| Mercereau et.al[45] | Yes | Yes | NA | Yes | NA | Yes | Yes | Yes | Yes | Yes |
| Uejio et.al[52] | Yes | Yes | NA | Yes | NA | Yes | Yes | Yes | Yes | Yes |
| Voelkel et.al[53] | Yes | Yes | NA | Yes | NA | Yes | Yes | Yes | Yes | Yes |
| Hansen et.al[46] | Yes | Yes | Yes | Yes | Yes | Yes | Yes | Yes | Yes | Yes |
